# Prevalence of Varroa Mite and Associated Viruses in *Apis mellifera jemenitica* in Arid and Semi-Arid Regions

**DOI:** 10.3390/insects17070663

**Published:** 2026-06-25

**Authors:** Yehya Alattal, Khaled El-Asha, Ahmad Alghamdi

**Affiliations:** Department of Plant Protection, College of Food and Agriculture Sciences, King Saud University, Riyadh 11451, Saudi Arabia

**Keywords:** DWV, colony losses, Arabian honey bee, ecological resistance

## Abstract

The Arabian honeybee, *Apis mellifera jemenitica*, is well adapted to the arid and semi-arid conditions of the Arabian Peninsula. However, little is known about the interactions among climatic conditions, Varroa mite, and honeybee viruses affecting *A. m. jemenitica* colonies. This study investigated the prevalence of *Varroa destructor* and major honey bee viruses (DWV, BQCV, SBV, CBPV, ABPV, KBV, IAPV and AIV) in *A. m. jemenitica* colonies located across arid and semi-arid regions of Saudi Arabia. The results revealed a high prevalence of *Varroa* infestation and DWV in both climatic zones. The other viruses (BQCV, SBV, CBPV, ABPV, KBV, IAPV and AIV) showed significant spatial variation in prevalence across the surveyed regions. *Varroa* infestation levels were generally low and significantly higher in semi-arid environments compared to arid regions (*p* < 0.001). Mean regional DWV loads (copies/bee) were consistently low across all *A. m. jemenitica* colonies (6.8 × 10^2^ to 4.4 × 10^3^ copies/bee), were positively linked with *Varroa* infestation levels (R^2^ = 0.63), and demonstrated significant variation among climatic zones (*p* < 0.001). Virus co-occurrence analysis using Phi (ϕ) coefficients revealed structured viral communities, with several virus pairs exhibiting moderate to strong positive associations. Overall, this study highlights the low DWV loads in *A. m. jemenitica* colonies, and the impact of climate conditions on Varroa mite–virus interactions in shaping honeybee health under arid and semi-arid conditions.

## Simple Summary

Honey bee colonies worldwide face major threats from parasites and viral diseases, with the Varroa mite being one of the most damaging pests. This study examined *Varroa* infestation levels and the occurrence of important honey bee viruses (DWV, BQCV, SBV, CBPV, ABPV, KBV, IAPV and AIV) in the native Arabian honey bee, *Apis mellifera jemenitica*, across arid and semi-arid regions of Saudi Arabia. Varroa mites and Deformed Wing Virus (DWV) were dominant in the surveyed colonies. Results showed that colonies with higher *Varroa* infestation generally carried higher levels of DWV, highlighting the important role of the mite in spreading DWV infections. The study also showed spatial variation in the prevalence of major honeybee viruses between arid and semi-arid regions. Despite the widespread presence of *Varroa* and viruses, DWV levels remained relatively low, suggesting that the Arabian honey bee may possess natural adaptations that keep DWV levels low. These findings improve our understanding of honey bee health in arid environments and may support the development of management strategies tailored to local conditions.

## 1. Introduction

The ectoparasitic mite *Varroa destructor* is widely recognized as the most destructive pest of *Apis mellifera* colonies worldwide. It weakens colonies, transmits multiple viruses, and can ultimately lead to colony collapse if left unmanaged [1,2,3,4,5,6,7]. However, *A. mellifera* subspecies exhibit differing responses to varroasis, reflecting variation in behavioral, physiological, and genetic traits, as well as environmental adaptation [8,9,10,11]. In contrast to many African honey bee subspecies, such as *A. m. scutellata*, European subspecies including *A. m. ligustica* and *A. m. carnica* are generally more susceptible to *Varroa* infestation and often require intensive management interventions [8,10,12].

In addition to subspecies-level variation in resistance, the interaction between *A. mellifera* colonies and *V. destructor* varies significant across climatic regions. In cooler climates, prolonged brood-rearing periods and seasonal colony dynamics favor sustained mite reproduction, resulting in high infestation levels and increased transmission of viruses, particularly Deformed Wing Virus (DWV), which is strongly associated with winter colony losses [8,13,14,15,16]. Furthermore, overwintering stress in Northern Europe exacerbates the combined effects of *Varroa* infestation and viral infections, often resulting in elevated mortality rates if colonies are not intensively managed [4,17,18,19,20].

In contrast, arid and semi-arid environments are characterized by extreme temperatures, reduced brood production, and smaller colony sizes, factors that may limit *Varroa* population growth and slow disease progression [8,21,22]. However, these environments may also impose physiological stress on colonies, increasing susceptibility to infection during periods of resource scarcity or climatic extremes. Consequently, viral prevalence may exhibit episodic increases rather than sustained high levels [23,24,25]. Transitional semi-arid zones often display the greatest variability in viral dynamics due to strong seasonal fluctuations in host population size, brood availability, and vector pressure.

The Arabian honey bee, *A. m. jemenitica*, is the indigenous subspecies of the Arabian Peninsula and is highly adapted to arid and semi-arid environments [26,27,28,29]. This subspecies generally exhibits lower *V. destructor* infestation levels than *A. m. carnica* and *A. m. ligustica* under comparable environmental conditions [12,30]. Reduced infestation levels have not been primarily attributed to impaired mite reproduction or infertility, but rather to colony-level traits such as smaller colony size, reduced brood area, and enhanced grooming and hygienic behaviors [27,31]. Furthermore, high ambient temperatures may reduce mite survival and increase natural mite fall, potentially contributing to lower infestation levels [8,32,33]. Collectively, these findings suggest that resistance to *V. destructor* is a multifactorial trait influenced by both host genetic background and environmental conditions [34].

Beyond its direct effects on colony health, *V. destructor* is the primary biological vector of honey bee viruses and plays a central role in the transition of infections from covert to overt states [13,14]. Approximately 22 viruses have been reported to infect *Apis* species worldwide [35]. Among these, Deformed Wing Virus (DWV), Kashmir Bee Virus (KBV), Acute Bee Paralysis Virus (ABPV), Chronic Bee Paralysis Virus (CBPV), Apis iridescent virus (AIV), and Israeli Acute Paralysis Virus (IAPV) are strongly associated with *Varroa*-mediated transmission [35,36,37,38]. These viruses are widely distributed and constitute some of the most important viral pathogens affecting honey bee health. Following infection, viruses may spread through both horizontal transmission (e.g., trophallaxis and direct contact) and vertical transmission via infected queens [39,40].

In contrast, Black Queen Cell Virus (BQCV) and Sacbrood Virus (SBV) primarily infect immature developmental stages and are transmitted mainly through glandular secretions, contaminated food, and pollen. Although *V. destructor* has occasionally been reported as a carrier of these viruses, it is not considered a major biological vector in their epidemiology [41,42].

Due to shared transmission routes and common epidemiological drivers, viral co-occurrence is widespread in *A. mellifera* colonies. Multiple viruses frequently co-infect individual bees and colonies, forming complex pathogen communities [19,37,43,44]. *V. destructor* plays an important role in facilitating these interactions, particularly those involving DWV, by increasing opportunities for virus transmission and altering host–pathogen dynamics [45,46,47,48,49]. While DWV often dominates the viral community, other viruses may persist at varying prevalences and abundances depending on environmental conditions, colony health, and host genetic background [19,50,51].

Variation in susceptibility to DWV among *A. mellifera* subspecies has been widely reported. European subspecies such as *A. m. carnica* and *A. m. ligustica* often exhibit high viral loads (>10^6^ copies/bee) and severe clinical symptoms under conditions of substantial *Varroa* infestation [10,18,52,53]. In contrast, African honey bee populations frequently maintain lower DWV loads and experience fewer overt symptoms, although the mechanisms underlying these patterns remain incompletely understood [54,55]. Proposed explanations include differences in host behavior, colony demography, mite infestation levels, and environmental adaptation. In addition, adaptation to hot and arid environments has been suggested as a factor that may reduce the impact of viral infections, although direct evidence remains limited [56,57,58].

Across climatic zones, variation in viral prevalence and abundance reflects the combined influence of ecological and epidemiological factors rather than climate alone. Host susceptibility, *Varroa* population dynamics, colony management, resource availability, and environmental stressors collectively shape viral communities and disease outcomes [23,25,59,60].

Therefore, this study aimed to investigate *V. destructor* infestation levels and the prevalence, distribution, and co-occurrence of major honey bee viruses in *A. m. jemenitica* colonies across arid and semi-arid regions of Saudi Arabia. Specifically, the study examined the occurrence of Deformed Wing Virus (DWV), Black Queen Cell Virus (BQCV), Sacbrood Virus (SBV), Kashmir Bee Virus (KBV), Acute Bee Paralysis Virus (ABPV), Chronic Bee Paralysis Virus (CBPV), Apis Iridescent Virus (AIV), and Israeli Acute Paralysis Virus (IAPV), and evaluated their distribution patterns under contrasting environmental conditions. We hypothesized that (i) virus prevalence and diversity differ between arid and semi-arid regions, (ii) higher *V. destructor* infestation levels are associated with increased prevalence of major honey bee viruses, particularly DWV, and (iii) environmental conditions influence viral co-occurrence patterns within honey bee colonies. By integrating pathogen surveillance with environmental context, this study provides new insights into the ecological drivers of virus dynamics and their implications for honey bee health in arid and semi-arid ecosystems.

## 2. Materials and Methods

### 2.1. Sampling

**Figure 1:**
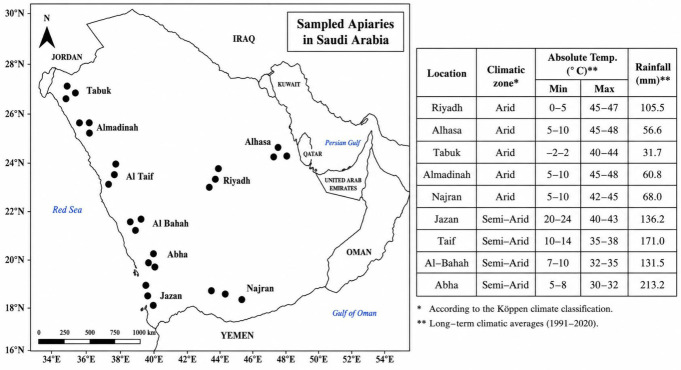
Location of sampled apiaries within Saudi Arabia and the climatic zone, annual maximum and minimum temperatures, and average annual precipitation of each location (National Center of Metrology; https://www.ncm.gov.sa, accessed on 18 December 2025).

Samples were collected from nine beekeeping regions in Saudi Arabia (Abha, Jazan, Taif, Almadinah, Albaha, Riyadh, Tabuk, and Alhasa), representing two climatic zones: an arid zone characterized by high maximum temperatures (42–47 °C) and a semi-arid zone with comparatively milder temperatures (30–38 °C) (Figure 1). Within each region, three non-migratory apiaries of the indigenous honey bee, *A. m. jemenitica*, were selected, and three colonies were sampled from each apiary. This sampling design yielded nine colonies per region and a total of 81 colonies across the study area.

For each colony, approximately 100 adult worker bees were collected from the brood chamber, pooled, and processed as a single sample for pathogen detection and quantification. No colony contributed more than one sample to the survey. Consequently, viral loads represent colony-level estimates rather than individual-level measurements, and variation among individual bees within colonies could not be assessed. Because colonies were sampled within apiaries and regions, the study design was hierarchical. However, the statistical analyses were conducted at the colony level and did not explicitly model apiary- or region-level random effects. Therefore, the results should be interpreted primarily as colony-level and regional patterns of pathogen prevalence and abundance.

The selected apiaries had not received any *V. destructor* treatments for at least eight months prior to sampling. Samples were preserved in 50 mL vials containing RNAlater^TM^ solution (ThermoFisher Scientific Inc., Waltham, MA, USA), transported under cooled conditions, and subsequently stored at −20 °C until further processing. These samples were used for both *Varroa* infestation assessment and DNA/RNA extraction.

To confirm subspecies identity, an additional sample of 20 worker bees was collected from each colony. Subspecies affiliation was determined using IdentiFly v1.8 (https://www.drawwing.org/identifly, accessed on 15 January 2026) based on geometric morphometric analysis of wing venation patterns. Briefly, the right forewing of each worker bee was dissected, cleaned, mounted on a microscope slide, and photographed. Landmark-based morphometric analysis was then performed using IdentiFly v1.8, whereby key wing venation landmarks were digitized and analyzed. The resulting shape variables were compared with reference datasets incorporated within the software to assign specimens to subspecies groups.

### 2.2. Determination of Varroa Infestation Level

To determine *V. destructor* infestation levels, RNAlater^TM^ solution was first removed from each sample using a double-layer honey sieve. The bees were then washed in water containing a small amount of detergent and shaken for 2–3 min to dislodge attached mites. The contents of each vial were subsequently poured through the sieve, and a gentle jet of water was used to separate mites from adult bees. Recovered mites, including those remaining in the vials after washing, were counted, and infestation levels were calculated as the number of mites per 100 adult bees and expressed as a percentage [1,61].

Following mite quantification, the bees were re-immersed in RNAlater^TM^ solution and processed for nucleic acid extraction. For this purpose, the heads and thoraces of 100 worker bees from each colony were dissected, pooled, and used for DNA and RNA extraction. In total, 81 pooled colony samples were processed.

### 2.3. DNA/RNA Extraction and cDNA Synthesis

For the detection of Apis Iridescent Virus (AIV) and *Varroa destructor* 16S rRNA, genomic DNA was extracted using Chelex^®^ 100 Resin (Bio-Rad Laboratories, Hercules, CA, USA) according to the manufacturer’s instructions. The inclusion of *V. destructor* 16S rRNA primers was intended solely to confirm the presence of *Varroa* DNA in bee samples, which may have originated from attached mites or mite-derived material introduced during feeding. Quantification of *Varroa* infestation levels, however, was based exclusively on manual mite counts obtained using the washing method described above.

For the detection of all other viruses, total RNA was extracted using the TRIzol^TM^ Plus RNA Purification Kit (Invitrogen, Carlsbad, CA, USA), followed by additional purification using RNeasy columns (Qiagen, Germantown, TN, USA). RNA integrity was verified by agarose gel electrophoresis, and RNA quality was assessed using a NanoDrop^TM^ 1000 spectrophotometer (Thermo Fisher Scientific, Wilmington, DE, USA) based on the A260/A280 absorbance ratio. Purified RNA samples were stored at −80 °C until further use.

First-strand cDNA was synthesized from 1 µg of total RNA per sample using the SuperScript^TM^ III First-Strand Synthesis SuperMix (Invitrogen, Carlsbad, CA, USA) according to the manufacturer’s instructions. Reverse transcription was performed using a combination of oligo(dT) and random primers. The resulting cDNA was subsequently used as a template for conventional polymerase chain reaction (PCR) (presence/absence) and quantitative real-time PCR (qPCR) assay for the quantification of Deformed Wing Virus (DWV).

### 2.4. Primers and PCR Amplification

Previously published primers were used for the detection of Black Queen Cell Virus (BQCV), Sacbrood Virus (SBV), Kashmir Bee Virus (KBV), Acute Bee Paralysis Virus (ABPV), Chronic Bee Paralysis Virus (CBPV), Apis Iridescent Virus (AIV), Israeli Acute Paralysis Virus (IAPV), and *V. destructor* 16S rRNA (Table 1). PCR amplification was performed using a commercial PCR Master Mix (Thermo Fisher Scientific Inc., Waltham, MA, USA) according to the manufacturer’s instructions. Amplified products were separated on 2% agarose gels, stained with GelRed^®^ (Biotium, Fremont, CA, USA), and visualized under UV illumination. Virus occurrence was recorded as either present or absent, based on the expected amplicon size.

Absolute Deformed Wing Virus (DWV) loads were quantified by quantitative real-time PCR (qPCR) using SYBR^®^ Green PCR Master Mix (Applied Biosystems, Carlsbad, CA, USA) on a 7500 Real-Time PCR System (Applied Biosystems, Carlsbad, CA, USA). Each 25 µL reaction contained 13 µL of SYBR^®^ Green Master Mix, 2 µL of forward primer (2 pmol), 2 µL of reverse primer (2 pmol) (Table 1), 2 µL of cDNA template, and 6 µL of nuclease-free water. The ribosomal protein 49 (*Rp49*) gene was used as an endogenous control. A no-template control and a serial dilution of a positive DWV standard were included in each run.

Thermal cycling conditions consisted of an initial denaturation at 95 °C for 5 min, followed by 40 cycles of 95 °C for 10 s, 57 °C for 30 s, and 72 °C for 10 s. Amplification specificity was assessed by melting curve analysis following qPCR amplification. Melting profiles were generated over a temperature range of 55–95 °C with 0.5 °C increments. Samples were considered positive when a single peak corresponding to the expected melting temperature (Tm) was observed, indicating specific amplification in the absence of detectable primer-dimers or non-specific products.

All reactions were performed in triplicate. Absolute quantification of DWV was achieved using a standard curve generated from serial dilutions of a DWV standard with a known copy number. Cycle threshold (Ct) values were converted to viral copy numbers using the standard curve and expressed as copies per bee (copies/bee). The standard curve exhibited excellent linearity (R^2^ > 0.99) with a slope of −3.33, corresponding to a linearity of approximately 99.7%.

### 2.5. Statistical Analysis

All statistical analyses were conducted to evaluate the effects of climatic zone (arid vs. semi-arid) on *V. destructor* infestation levels, pathogen prevalence, and Deformed Wing Virus (DWV) loads. The sampling design consisted of independent colonies within apiaries and apiaries within regions, which were subsequently grouped into two climatic zones: arid regions (Riyadh, Alhasa, Almadinah, Najran and Tabuk) and semi-arid regions (Abha, Albaha, Jazan, and Taif). For all analyses, the colony was considered the primary biological sampling unit because viral occurrence and Deformed Wing Virus (DWV) load were determined independently for each colony sample. The study included nine regions, three apiaries per region, and three colonies per apiary, yielding a total of 81 colony samples.

Prior to analysis, data normality was assessed using the Shapiro–Wilk test, and homogeneity of variances was evaluated using Levene’s test. To compare *Varroa* infestation levels and DWV loads between climatic zones, independent-samples *t*-tests were applied when assumptions of normality and homogeneity of variance were satisfied. When assumptions were not met, the non-parametric Mann–Whitney U test was used. Effect size was estimated using Cohen’s *d*, calculated as the difference between group means divided by the pooled standard deviation.

For pathogen prevalence (presence/absence data), differences between climatic zones were assessed using the chi-square test of independence. Fisher’s exact test was applied when expected cell frequencies were less than five. Differences in pathogen community composition between climatic zones were evaluated using permutational multivariate analysis of variance (PERMANOVA) based on Bray–Curtis dissimilarities with 999 permutations.

Regional differences in *Varroa* infestation levels and DWV loads were assessed using one-way analysis of variance (ANOVA), followed by Tukey’s Honestly Significant Difference (HSD) test for multiple pairwise comparisons when significant effects were detected (*p* < 0.05). Associations between *Varroa* infestation levels and DWV loads were evaluated using Spearman’s rank correlation coefficient (ρ). Statistical analyses were performed using R software (R Foundation for Statistical Computing, Vienna, Austria), 7500 Real-Time PCR Software v2.0.6 (Applied Biosystems, Foster City, CA, USA), and Microsoft Excel. Statistical significance was set at *p* < 0.05.

## 3. Results

This study represents the first investigation of the prevalence and load of Deformed Wing Virus (DWV) and other honey bee viruses associated with *V. destructor* infestation in *A. m. jemenitica* colonies. *V. destructor* was detected in all surveyed colonies, resulting in a prevalence of 100% across both arid and semi-arid regions. However, infestation levels differed significantly between climatic zones, with semi-arid regions exhibiting higher infestation levels than arid regions (Welch’s *t*-test, *p* < 0.001; Mann–Whitney U test, *p* < 0.001). Mean regional infestation levels ranged from 5.0 ± 2.1% to 28.0 ± 8.0%, with the highest values observed in Jazan, Abha, and Albaha. In contrast, colonies from Riyadh, Almadinah, Alhasa, and Tabuk consistently exhibited lower infestation levels. The large effect size (Cohen’s *d* > 1.5) indicates a strong association between climatic zone and *Varroa* infestation intensity.

**Figure 2:**
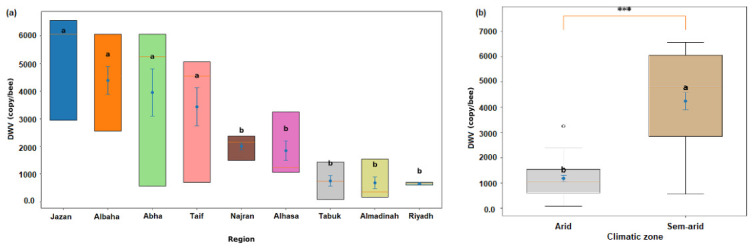
(**a**) Distribution of DWV load (copies per bee) among sampled regions of Saudi Arabia. Boxplots show the median (horizontal line) and interquartile range (box), and the square marker indicates the mean value. Significant differences among regional means were calculated according to Tukey’s honestly significant difference (HSD) post hoc test following one-way ANOVA (*p* < 0.05). (**b**) Comparison of virus load between arid and semi-arid climatic zones. Boxplots represent the median (horizontal line), circular markers indicate the mean virus load, and error bars represent the standard error of the mean (SE). Different lowercase letters indicate significant differences between climatic zones, while asterisks (***) denote highly significant differences (*p* < 0.001). Honey bee colonies from the semi-arid zone exhibited significantly higher virus loads than those from the arid zone, highlighting the influence of climatic conditions on viral infection intensity.

Deformed Wing Virus (DWV) was detected in all *A. m. jemenitica* colonies and generally occurred at relatively low loads (Figure 2). Mean regional DWV loads ranged from 681.8 ± 749.3 to 4389.0 ± 1756.6 copies per bee, although substantial variation was observed among colonies. Similar to *Varroa* infestation, DWV loads were significantly higher in semi-arid regions than in arid regions (Welch’s *t*-test, *p* < 0.001; Mann–Whitney U test, *p* < 0.001). Colonies from semi-arid regions exhibited both higher average viral loads and greater variability, whereas colonies from arid regions generally maintained lower DWV loads, with most samples clustering at the lower end of the distribution (Figure 2).

**Figure 3:**
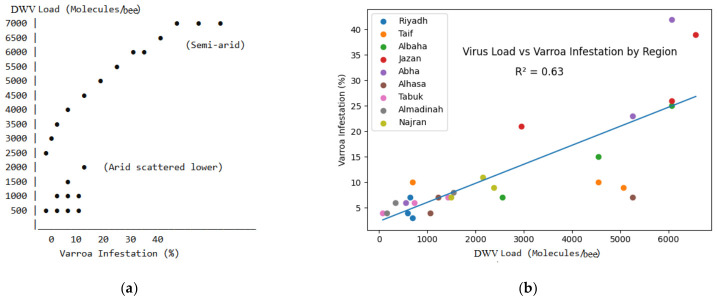
(**a**) Relationship between *Varroa* mite infestation (%) and DWV load (copies/bee) under arid and semi-arid conditions. Each point represents an individual Apiary. (**b**) Colored points represent an individual apiary, with colors indicating regions including Riyadh, Taif, Albaha, Jazan, Abha, Alhasa, Tabuk, Almadinah, and Najran (right). The fitted linear regression line indicates a positive association between DWV load and *Varroa* mite infestation (R^2^ = 0.63), suggesting that increased mite infestation is associated with higher DWV titers.

Regression analysis further demonstrated that climatic zone and *V. destructor* infestation were both significantly associated with variation in DWV load (Figure 3). The fitted model explained a substantial proportion of the observed variation (R^2^ = 0.63). Moreover, the significant interaction between climatic zone and *Varroa* infestation (*p* < 0.05) indicated that the relationship between mite infestation and DWV load differed between environments. Specifically, the positive association between infestation intensity and DWV load was stronger in semi-arid regions than in arid regions, suggesting that environmental conditions may influence the strength of the relationship between *Varroa* infestation and viral abundance.

**Figure 4:**
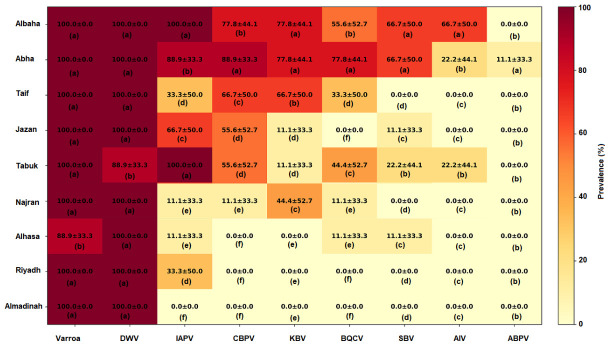
Heatmap showing the regional prevalence (%) of *Varroa* mites and investigated honeybee viruses across arid and semi-arid regions of Saudi Arabia. Cell colors represent virus prevalence based on the proportion of positive samples detected within each region (*n*= 9 colonies per region), Values within cells are presented as mean prevalence (%) ± standard deviation. Different lowercase letters within each virus column indicate significant differences among regions according to pairwise Fisher’s exact tests (*p* < 0.05).

Marked regional differences were observed in the prevalence and diversity of honey bee viruses (Figure 4). DWV was the most prevalent virus and was detected in nearly all surveyed regions, indicating widespread occurrence across contrasting environmental conditions. In contrast, BQCV, SBV, CBPV, IAPV, KBV, and AIV occurred at lower frequencies and exhibited greater regional variability, whereas ABPV was detected only sporadically.

Semi-arid regions, particularly Albaha and Abha, exhibited the highest viral diversity and co-infection frequencies, with multiple viruses commonly detected within the same colonies. Jazan also displayed relatively high pathogen richness. In contrast, the arid regions of Riyadh, Almadinah, and Alhasa were characterized by lower viral diversity and a predominance of DWV. The greater viral diversity observed in semi-arid regions suggests that environmental conditions may influence pathogen community structure and the occurrence of multi-virus infections within colonies.

Analysis of virus co-occurrence patterns using Phi (ϕ) coefficients and Fisher’s exact tests revealed several significant positive associations among pathogens (Table 2). The strongest association was observed between *V. destructor* infestation and DWV occurrence (ϕ > 0.9, *p* < 0.001), consistent with the well-established role of *Varroa* as a major vector of DWV. In addition, IAPV, CBPV, BQCV, and SBV formed a cluster of positively associated viruses that frequently co-occurred within colonies (*p* < 0.01). These associations may reflect shared transmission routes, common epidemiological drivers, or similar ecological requirements for persistence within colonies. In contrast, ABPV showed no significant associations with other pathogens, while several virus pairs exhibited weak or negligible correlations, suggesting largely independent occurrence patterns. Collectively, these findings indicate that honey bee colonies harbor complex pathogen communities in which virus distribution is influenced by both vector-mediated transmission and environmental factors.

## 4. Discussion

The DWV loads observed in *A. m. jemenitica* colonies (10^2^–10^3^ copies/bee) were substantially lower than those commonly reported in European honey bee populations, where viral loads frequently reach 10^6^–10^9^ copies/bee and may exceed 10^10^ copies/bee in heavily infected colonies. Similar high viral burdens have been associated with *V. destructor* infestation, clinical symptoms, and overwintering colony losses in European *A. mellifera* populations [65,66]. DWV loads were also lower than those reported in Africanized honey bees from Brazil and African honey bees from South Africa [10,54,55]. These observations suggest differences in host–pathogen dynamics in *A. m*. *jemenitica*, potentially reflecting ecological adaptation, reduced viral amplification, or other factors associated with local environmental conditions. However, the present study did not directly assess viral resistance or tolerance. Comparative studies incorporating colony health, survival, productivity, and physiological responses are therefore required to determine whether these relatively low viral loads are associated with enhanced resilience or other host-related mechanisms.

The higher *V. destructor* infestation levels observed in semi-arid regions were associated with environmental conditions that may favor mite persistence and reproduction, including moderate temperatures, relatively higher humidity, and extended brood availability [22,23,67,68,69,70,71,72]. Semi-arid regions also exhibited higher DWV loads, consistent with the well-established association between *Varroa* infestation and virus transmission. Previous studies have shown that increased mite densities are frequently associated with greater viral prevalence and abundance within colonies [73,74,75]. The patterns observed in the present study are consistent with these findings.

The regression model further demonstrated that both climatic zone and *Varroa* infestation were significantly associated with variation in DWV load. In addition, the significant interaction term (*p* < 0.05) indicated that the relationship between mite infestation and DWV load differed between climatic zones. Specifically, the association between infestation intensity and DWV load was stronger in semi-arid regions than in arid regions. This observation is consistent with the possibility that environmental conditions influence the relationship between *Varroa* infestation and viral abundance. Similar interactions among climate, host, parasite, and pathogen dynamics have been reported in previous studies of honey bee health. The variability observed among colonies within the same climatic zone further suggests that local environmental conditions, colony management practices, and host genetic background may contribute to pathogen dynamics.

Several viruses, including ABPV, AIV, and KBV, demonstrated lower prevalence or sporadic detection in the surveyed colonies. Their low prevalence may reflect limited circulation within local honey bee populations, episodic introductions, or ecological conditions that are less favorable for sustained transmission. Long-term monitoring would help determine whether these viruses represent transient infections or components of a stable, but low-prevalence viral community.

Acute Bee Paralysis Virus (ABPV) was only rarely detected despite the widespread occurrence of *V. destructor*. This finding contrasts with observations from some European honey bee populations, where ABPV is more frequently associated with *Varroa* infestation [41,76,77,78,79]. The extremely low prevalence observed in the present study suggests that ABPV is either absent or circulating at very low levels within *A. m. jemenitica* populations. Similar patterns have been reported in African and Middle Eastern honey bee populations, where DWV often dominates the viral community while ABPV remains uncommon [80,81]. The mechanisms underlying this pattern remain unclear but may involve differences in host susceptibility, viral competition, vector competence, or environmental conditions.

Analysis of virus co-occurrence patterns revealed both positive and weak associations among honey bee pathogens. The positive associations observed among several viruses commonly linked with *V. destructor* are consistent with shared transmission pathways and common epidemiological drivers. In contrast, weak or near-zero Phi (ϕ) coefficients suggest largely independent occurrence patterns among some virus pairs. Semi-arid regions exhibited greater viral diversity and more frequent co-occurrence of viruses than arid regions, indicating that environmental conditions may influence pathogen community structure. However, the mechanisms underlying these patterns were not directly investigated and warrant further study.

Overall, *A. m. jemenitica* colonies maintained relatively low DWV loads despite the widespread occurrence of *V. destructor* across contrasting climatic zones. The observed associations among climatic conditions, *Varroa* infestation intensity, and DWV load highlight the potential importance of environmental factors in shaping honey bee disease dynamics. However, because this study was observational, the underlying mechanisms remain unresolved. Additional experimental and longitudinal studies incorporating colony health, survival, productivity, and physiological traits are needed to determine whether the observed patterns reflect enhanced resilience, reduced susceptibility, or other adaptive responses of *A. m. jemenitica* to viral infection.

## Figures and Tables

**Table 1:** List of primer/probe sets used for the detection of bee viruses in *A. m. jemenitica*.

Target	Primer Name	Sequence (5′–3′)	Amplicon (bp)	Reference
Kashmir Bee Virus	KBV83F	ACCAGGAAGTATTCCCATGGTAAG	~79	[62]
	KBV161R	TGGAGCTATGGTTCCGTTCAG		
Acute Bee Paralysis Virus	ABPV95F	TCCTATATCGACGACGAAAGACAA	~65	[62]
	ABPV159R	GCGCTTTAATTCCATCCAATTGA		
Chronic Bee Paralysis Virus	CBPV304F	TCTGGCTCTGTCTTCGCAAA	~1025	[62]
	CBPV371R	GATACCGTCGTCACCCTCATG		
Deformed Wing Virus	DWV958F	CCTGGACAAGGTCTCGGTAGAA	~110	[62]
	DWV9711R	ATTCAGGACCCCACCCAAAT		
Apis Iridescent Virus	AIV12F	GGCTAGTAAACGTAGTGGATATGACAAT	~95	[62]
	AIV106R	CAC CTGGTGGTCCAAGAGAAG		
Black Queen Cell Virus	BQCV8195F	GGTGCGGGAGATGATATGGA	~170	[62]
	BQCV8365R	GCCGTCTGAGATGCATGAATAC		
Sac Brood Virus	SBV311F	AAGTTGGAGGCGCGYATTTG	~100	[62]
	SBV380R	CAAATGTCTTCTTACDAGAAGYAAGGATTG		
*V. destructor* 16S rRNA	*Varroa* 16S 12290F	ATTACGTCGGTCTGAACTCAAA	~108	[63]
	*Varroa* 16S 12398R	TTGCGACCTCGATGTTGAATT		
Israeli Acute Paralysis Virus	IAPV-F	GCGGAGAATATAAGGCTCAG	~100	[64]
	IAPV-R	CTTGCAAGATAAGAAAGGGGG		
Deformed wing virus (Probe)	DWV9627T	FAM-CATGCTCGAGGATTGGGTCGTCGT-TAMARA		[62]

**Table 2:** Pairwise associations among *Varroa* mite and eight honeybee viruses ((DWV, IAPV, CBPV, KBV, BQCV, SBV, AIV, ABPV) across arid and semi-arid regions of Saudi Arabia using Phi coefficient (φ) values.

	*Varroa*	DWV	IAPV	CBPV	KBV	BQCV	SBV	AIV	ABPV
*Varroa*	1.00	0.98	0.45	0.32	0.28	0.30	0.27	0.20	0.05
DWV	0.98	1.00	0.46	0.33	0.29	0.31	0.28	0.21	0.05
IAPV	0.45	0.46	1.00	0.58	0.42	0.50	0.48	0.36	0.02
CBPV	0.32	0.33	0.58	1.00	0.44	0.52	0.49	0.35	0.01
KBV	0.28	0.29	0.42	0.44	1.00	0.47	0.41	0.33	0.00
BQCV	0.30	0.31	0.50	0.52	0.47	1.00	0.55	0.38	0.01
SBV	0.27	0.28	0.48	0.49	0.41	0.55	1.00	0.36	0.01
AIV	0.20	0.21	0.36	0.35	0.33	0.38	0.36	1.00	0.00
ABPV	0.05	0.05	0.02	0.01	0.00	0.01	0.01	0.00	1.00

## Data Availability

Data are available in the tables and figures.
